# A Traditional Chinese Medicine Syndrome Classification Model Based on Cross-Feature Generation by Convolution Neural Network: Model Development and Validation

**DOI:** 10.2196/29290

**Published:** 2022-04-06

**Authors:** Zonghai Huang, Jiaqing Miao, Ju Chen, Yanmei Zhong, Simin Yang, Yiyi Ma, Chuanbiao Wen

**Affiliations:** 1 College of Medical Information Engineering Chengdu University of Traditional Chinese Medicine Chengdu China; 2 School of Mathematics Southwest Minzu University Chengdu China; 3 College of Acupuncture-Moxibustion and Tuina Chengdu University of Traditional Chinese Medicine Chengdu China

**Keywords:** intelligent syndrome differentiation, cross-FGCNN, TCM

## Abstract

**Background:**

Nowadays, intelligent medicine is gaining widespread attention, and great progress has been made in Western medicine with the help of artificial intelligence to assist in decision making. Compared with Western medicine, traditional Chinese medicine (TCM) involves selecting the specific treatment method, prescription, and medication based on the dialectical results of each patient’s symptoms. For this reason, the development of a TCM-assisted decision-making system has lagged. Treatment based on syndrome differentiation is the core of TCM treatment; TCM doctors can dialectically classify diseases according to patients’ symptoms and optimize treatment in time. Therefore, the essence of a TCM-assisted decision-making system is a TCM intelligent, dialectical algorithm. Symptoms stored in electronic medical records are mostly associated with patients’ diseases; however, symptoms of TCM are mostly subjectively identified. In general electronic medical records, there are many missing values. TCM medical records, in which symptoms tend to cause high-dimensional sparse data, reduce algorithm accuracy.

**Objective:**

This study aims to construct an algorithm model compatible for the multidimensional, highly sparse, and multiclassification task of TCM syndrome differentiation, so that it can be effectively applied to the intelligent dialectic of different diseases.

**Methods:**

The relevant terms in electronic medical records were standardized with respect to symptoms and evidence-based criteria of TCM. We structuralized case data based on the classification of different symptoms and physical signs according to the 4 diagnostic examinations in TCM diagnosis. A novel cross-feature generation by convolution neural network model performed evidence-based recommendations based on the input embedded, structured medical record data.

**Results:**

The data set included 5273 real dysmenorrhea cases from the Sichuan TCM big data management platform and the Chinese literature database, which were embedded into 60 fields after being structured and standardized. The training set and test set were randomly constructed in a ratio of 3:1. For the classification of different syndrome types, compared with 6 traditional, intelligent dialectical models and 3 click-through-rate models, the new model showed a good generalization ability and good classification effect. The comprehensive accuracy rate reached 96.21%.

**Conclusions:**

The main contribution of this study is the construction of a new intelligent dialectical model combining the characteristics of TCM by treating intelligent dialectics as a high-dimensional sparse vector classification task. Owing to the standardization of the input symptoms, all the common symptoms of TCM are covered, and the model can differentiate the symptoms with a variety of missing values. Therefore, with the continuous improvement of disease data sets, this model has the potential to be applied to the dialectical classification of different diseases in TCM.

## Introduction

According to the 2019 edition of *Latest* *Global Medical Summary* released by the World Health Organization, TCM is evaluated as complementary and alternative medicine that can effectively prevent and treat a variety of diseases [1]. TCM has been tested and refined through thousands of years of medical practice, exerting extensive influence in East Asia and even in the world [2]. In clinical practice, the effectiveness of TCM has been significant for chronic diseases such as chronic obstructive pulmonary disease [3] and diabetes [4] as well as for gynecological conditions such as infertility [5] and dysmenorrhea [6]. However, TCM, an empirical product, lacks objective evaluation indicators. The treatment process of TCM is more like a black box, which makes people doubt its reliability, but TCM does manifest advantages in clinical practice. Similarly, the result of neural network classification is also a black box to obtain the practice of the process. Therefore, an increasing number of Chinese researchers have begun to apply a neural network to explore the treatment rules of TCM to further prove its objective effectiveness [7-9].

Syndrome differentiation is an important classification task. The treatment of diseases in TCM is subject to certain law: principle-methods-formulas-medicinal. The principle refers to the guidance of TCM theory. More specifically, under the guidance of TCM theory, patients’ syndromes can be differentiated according to their symptoms, preferred treatment method is identified, an appropriate prescription is selected, and medication is chosen. In addition, correlation of all 4 examinations is essential to TCM treatment. Overall, the symptoms can be obtained from 4 diagnostic methods: inspection, auscultation and olfaction, inquiry, and palpation. From the point of view of machine learning, TCM dialectics can be regarded as a complex model, whose input is the 4 diagnostic information aspects of the patient and output is the syndrome type. With the advancement of machine learning technology, researchers have devoted themselves to the construction of this model. As traditional machine learning methods, decision tree [10,11], K-nearest neighbor [12], Bayes [13,14], and support vector machine (SVM) [15,16] have been widely used in intelligent dialectical tasks. In existing reports, these algorithms show satisfactory classification performance under complete data sets. However, electronic medical record data often have a significant amount of missing data. Missing data in these 4 models is a difficult problem to solve. With the rise of deep learning, neural networks have been gradually applied to such tasks [17,18]. With the deepening of the model, the amount of imbalanced data for different syndrome types is becoming more and more prominent. Due to the existence of rare syndrome types and unbalanced data sets of model diseases in TCM dialectics, over-fitting problems may occur in deep neural networks (DNNs). With the deepening of research, some researchers have further improved the training of dialectical models from the aspect of training data preprocessing to obtain a better fitting degree. From the point of symptom preprocessing, the 4 diagnosis information aspects were divided into multiple dimensions according to different categories [19]. Starting from the syndrome type, some researchers divide the syndrome type into smaller syndrome type factors [20]. These optimization methods solve the problem of data normalization to a certain extent but require more data integrity. In short, although existing models can distinguish their corresponding data sets well, they have high requirements with respect to data. Sufficient and complete patient information is required. In the real world, there is bound to be many missing data in patient information acquisition. Therefore, a model that is closer to the real world and that can effectively distinguish high-dimensional sparse data is needed.

The input dimension in the click-through-rate (CTR) task is large and sparse. Factorization machines (FMs) [21,22] obtain the relationship between 2 features by performing the inner product of the weights of the 2 corresponding features and automatically carrying out feature engineering. However, FMs are limited to a second-order cross-product in feature selection, hindering automatic selection of high-dimensional features. To automatically extract higher-order feature combinations, deep FMs classify each bit feature of the input into a field [23] based on the original FM model and construct a DNN in parallel to obtain high-order nonlinear features [24]. It can learn both low-order and high-order features, but it can only learn 2-dimensional and 1 high-dimensional feature and its coverage is not strong. With the advent of deep & cross network (DCN) [25], multidimensional cross features can be learned at the same time by using a cross network instead of FM. The DNN part of deep FM and DCN pays more attention to the nonlinear high-dimensional features generated by global data, ignoring some local features. Feature generation by convolution neural network (FGCNN) [26] uses a convolution neural network to extract local features and combines the advantages of the original multilayer perceptron (MLP) for global feature extraction, which allows the high-dimensional features of the model to contain more information. The success of the CTR model in the binary classification of high-dimensional sparse data inspired us to construct an improved dialectical multiclassification model suitable for the high-dimensional sparse symptom data of TCM.

Dysmenorrhea refers to severe pain in the lower abdomen before or during menstruation, which greatly affects the work, study, and life of women [27,28]. According to the presence or absence of pathological pelvic diseases, dysmenorrhea can be divided into primary dysmenorrhea and secondary dysmenorrhea [29]. At present, the main treatment of dysmenorrhea is nonsteroidal anti-inflammatory drugs or oral contraceptives; however, these medicines exert adverse effects on metabolism and the digestive system [30]. TCM has proven to be associated with fewer adverse effects and to have a more remarkable curative effect on dysmenorrhea [31,32]. It is considered a safe and reliable alternative therapy for dysmenorrhea. In this paper, dysmenorrhea data were divided into fields according to the diagnosis module of TCM. A cross-FGCNN model was constructed, in which linear cross features were obtained by cross-layer and nonlinear high-dimensional features were generated by FGCNN. The contributions of this paper are as follows:

1) According to the thinking system of TCM diagnosis, a filed segmentation suitable for TCM dialectics was constructed so that the model can better fit different diseases.

2) Because of the large dimension and high sparsity of TCM symptom data, we used cross-layer to obtain multidimensional linear cross features and used FGCNN to obtain nonlinear high-dimensional features, including local and global features. As many features as possible were obtained from sparse data.

3) Training data and test data consisted of 4000 real dysmenorrhea clinical cases from Sichuan TCM big data management platform and 1273 dysmenorrhea cases from the Chinese literature database, so diversity of medical record data source was ensured. Two professional TCM doctors verified the data according to the relevant standards of TCM to ensure reliability of the data. To ensure the objectivity of this study, 6 traditional, intelligent dialectical models and 3 CTR models were selected and compared with our model in terms of accuracy, F1 score, confusion matrix, and log-loss.

The structure of the rest of this paper is as follows: in the second section, we introduced our data acquisition, processing methods, and overall model structure; in the third section, we showed the experimental results; and in the last section, we put forward our conclusions.

## Methods

### Overview

**Figure 1:**
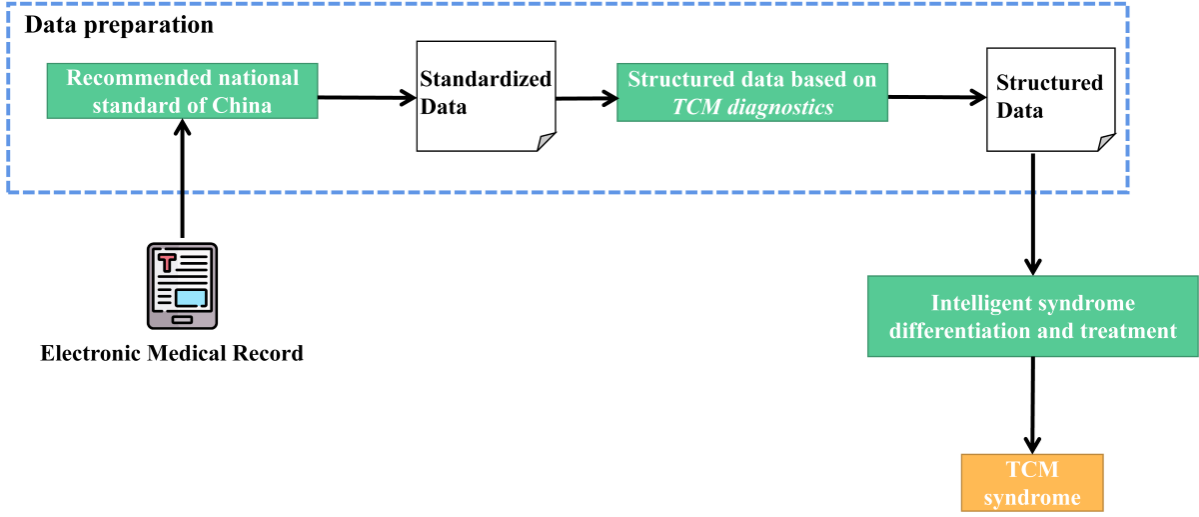
Intelligent syndrome differentiation block diagram. TCM: traditional Chinese medicine.

Figure 1 shows the intelligent syndrome differentiation block diagram. Electronic medical records were first standardized. Then, standardized data were structured according to the classification of symptoms and physical signs in TCM diagnostics. The first 2 steps were the data preparation module. Finally, the prepared data were input to the intelligent dialectical model for TCM dialectical classification.

### Data Preparation

A total of 4000 high-quality electronic medical records of dysmenorrhea were obtained from the Sichuan TCM big data management platform and 1273 cases of dysmenorrhea were obtained from the Chinese literature database. Any data related to the study were retained, such as symptoms, syndromes, and disease names. Before TCM doctors conduct syndrome differentiation, they synthesize the characteristics of many symptoms [28]. Therefore, in this study, professional TCM practitioners standardized the syndrome type and symptom dimension according to “GB/T20348-2006 TCM basic theory terminology” and “GB/T16751.2-1997 TCM Clinical diagnosis and treatment terminology-Syndrome part” issued by the State Administration of TCM. All symptoms related to the dialectics of TCM were classified according to the *Diagnostics of TCM*. For example, “white tongue coating” and “yellow tongue coating” were classified into inspection of tongue coating color. Later, the label encoder was carried out according to different symptom attributes. The overall division is shown in Table 1. Each symptom or physical sign represented an input dimension, so the overall input dimension was 60 dimensions. All symptoms or physical signs could not occur at the same time, so there must be a missing value and the missing value was −1.

**Table 1:** Classification of symptoms.

Diagnosis	Elements
Inspection (30 symptoms and physical signs in total)	Expression Complexion Physique Posture Head Face Nose Eye Ear Mouth Tooth Neck Chest Abdomen Lumbar Exterior genitalia Anus Skin Phlegm Saliva Vomitus Excrement Urinating Index fingers’ superficial venules Tongue nature Tongue shape Tongue color Tongue coating nature Tongue coating color Hypoglossal vessels
Listening and smelling (6 symptoms and physical signs in total)	Voice Breathing sound Snoring Coughing sound Belching Tone
Inquiry (22 symptoms and physical signs in total)	Cold and heat Sweating Pain site Nature of pain Head discomfort Physical discomfort Limb discomfort Ear discomfort Eye discomfort Sleep Diet Thirst Abnormal defecation Abnormal urine Menstrual period Menstrual color Menstrual volume Menstrual nature Emotion Family history Vaccination history Physiological abnormality
Pulse feeling and palpation (2 symptoms and physical signs in total)	Pulse condition Pressing feeling

According to our dysmenorrhea data, the main syndrome types could be summarized into 9 types: liver-kidney depletion, pattern of congealing cold with blood stasis, cold-dampness stagnation, liver constraint and dampness-heat, deficiency of qi and blood, qi stagnation and blood stasis, kidney deficiency and blood stasis, dampness-heat stasis obstruction, and yang deficiency and internal cold. Therefore, the output of our classification model only focused on these 9 syndrome types. The proportion of the 9 syndrome types is shown in Table 2, which shows that our data were unevenly distributed.

**Figure 2:**
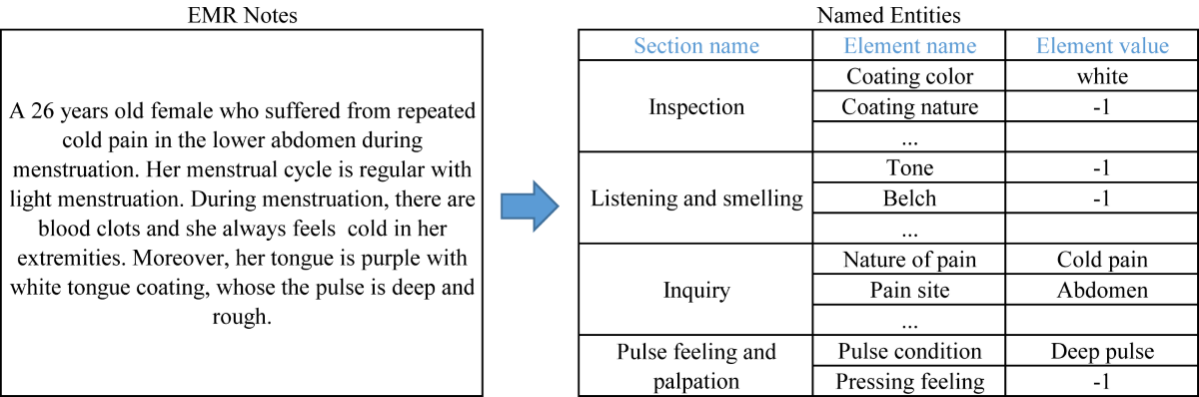
An example of electronic medical record preprocessing. EMR: electronic medical record.

Through standardization and structured operations, electronic medical data could be transformed into structured data. These data were used as input to the intelligent dialectical model. Figure 2 shows the preprocessing results of a real electronic medical record.

**Table 2:** Proportion of syndrome types (N=5273).

Syndrome type	Total, n (%)
Liver-kidney depletion	514 (9.7)
Pattern of congealing cold with stasis	720 (13.6)
Cold-dampness stagnation	568 (10.7)
Liver constraint and dampness-heat	522 (9.8)
Deficiency of qi and blood	575 (10.9)
Qi stagnation and blood stasis	751 (14.2)
Kidney deficiency and blood stasis	543 (10.2)
Dampness-heat stasis obstruction	544 (10.3)
Yang deficiency and internal cold	536 (10.1)

### Intelligent Syndrome Differentiation Model: Cross-FGCNN

First, this section illustrates an overview of cross-FGCNN, which can automatically learn the feature interaction of high-dimensional and sparse symptom data to complete the intelligent dialectical task. Next, we describe in detail how to extract high-order combination features of low-dimensional representation from high-dimensional sparse vectors. Finally, data are classified.

#### Overall Structure of the Model

**Figure 3:**
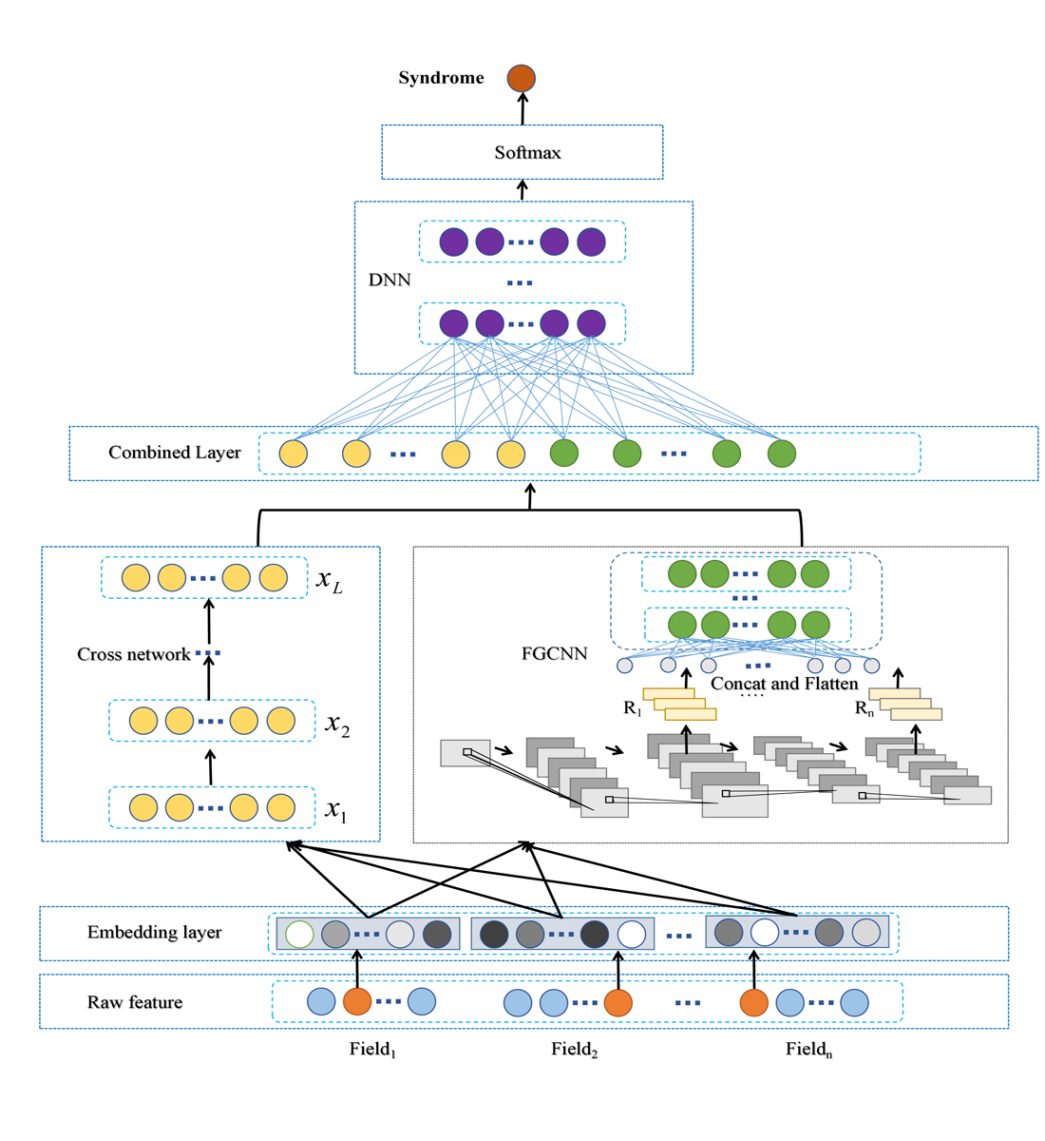
Model structure. DNN: deep neural network; FGCNN: feature generation by convolution neural network.

The whole model could be divided into 4 modules: data embedding module, cross linear feature extraction module, nonlinear feature extraction module, and classification module. The model read the symptom data found by label encoder and conducted a one-hot encoder according to each field. Then the embedding layer was used to map the high-dimensional sparse data to the low-dimensional dense features. The embedding data were used as shared input for the 2 parallel modules: the linear feature extraction module and the nonlinear feature extraction module. After the corresponding features were generated by the 2 modules, the 2 features were combined and input into the classification module, and finally the result syndrome type was obtained. The overall structure of the model is shown in Figure 3.

#### Data Embedding Module

**Figure 4:**
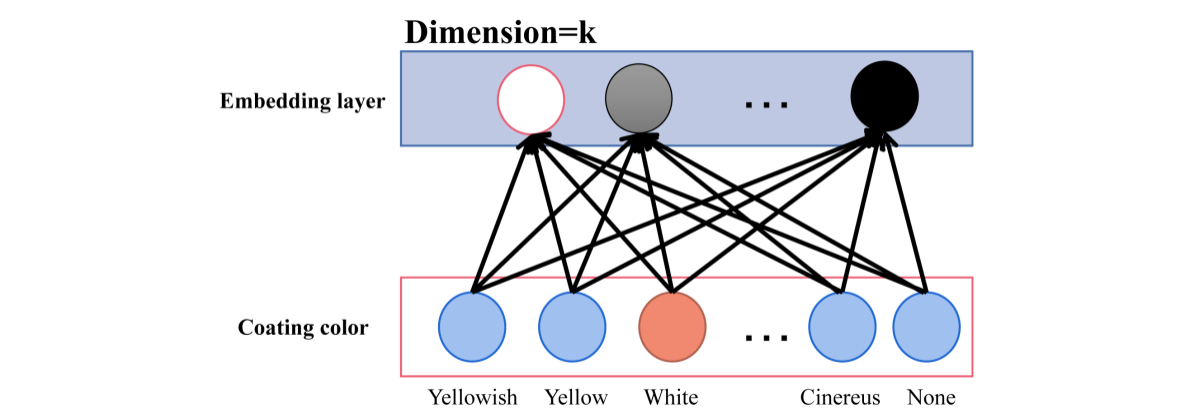
Coating color field of data embedding module.

The data embedding module of the model consisted of many structures as shown in Figure 4. According to the symptom and physical signs classification in *TCM diagnostics*, the obtained symptoms were mapped to different fields and one-hot coding was carried out. Embedding in the vector through dense embedding aimed to reduce the dimension of the embedding vector mapped from the field to the input model and ensured the density of the vector. For example, the physical sign, white tongue coating, was obtained from electronic cases and mapped to coating color field for encoding. The dimension of each field could be reduced to the specified dimension by the embedding operation, and the dimension of each field in the embedding layer was the same.

#### High-Order Linear Cross Feature Extraction Based on Cross Network

**Figure 5:**
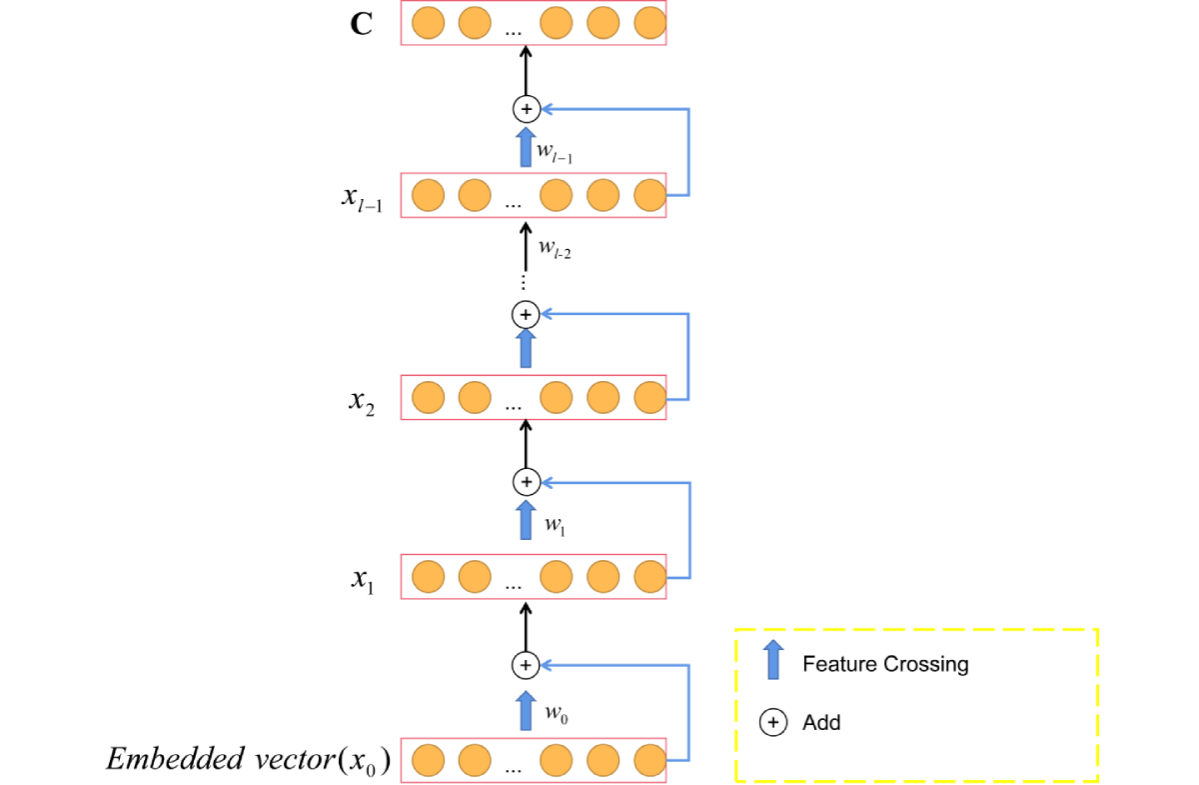
Cross network.

As shown in Figure 5, we used cross network to extract linear cross features in the model. As the number of layers increases, more cross features can be obtained. The output x_l+1_ or the layer l was obtained (1) from the original input data x_0_ and the output x_l_ from the previous layer. f(x_l_,w_l_,b_l_) was represented by (2), where b_l_ represents bias, w_l_ represents weight, x_0_ represents the initial input vector, and x_l_ represents the output vector of the upper layer. The advantage of (2) is that the input and output dimensions of each layer are the same while retaining the initial characteristics in each layer operation. Finally, the cross-feature vector C was obtained. Higher-order linear mixed features were obtained through the initial input vector and the previous output vector cross. The output of the previous layer was added after feature crossing, which is similar to residual and could effectively prevent gradient dissipation. As the number of layers of cross network increased, the degree of feature crossover in different fields increased.

x_l+1_ = f(x_l_,w_l_,b_l_)+x_l_ (1)

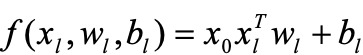
 (2)

#### Multiorder Nonlinear Cross Feature Extraction Based on FGCNN Module

**Figure 6:**
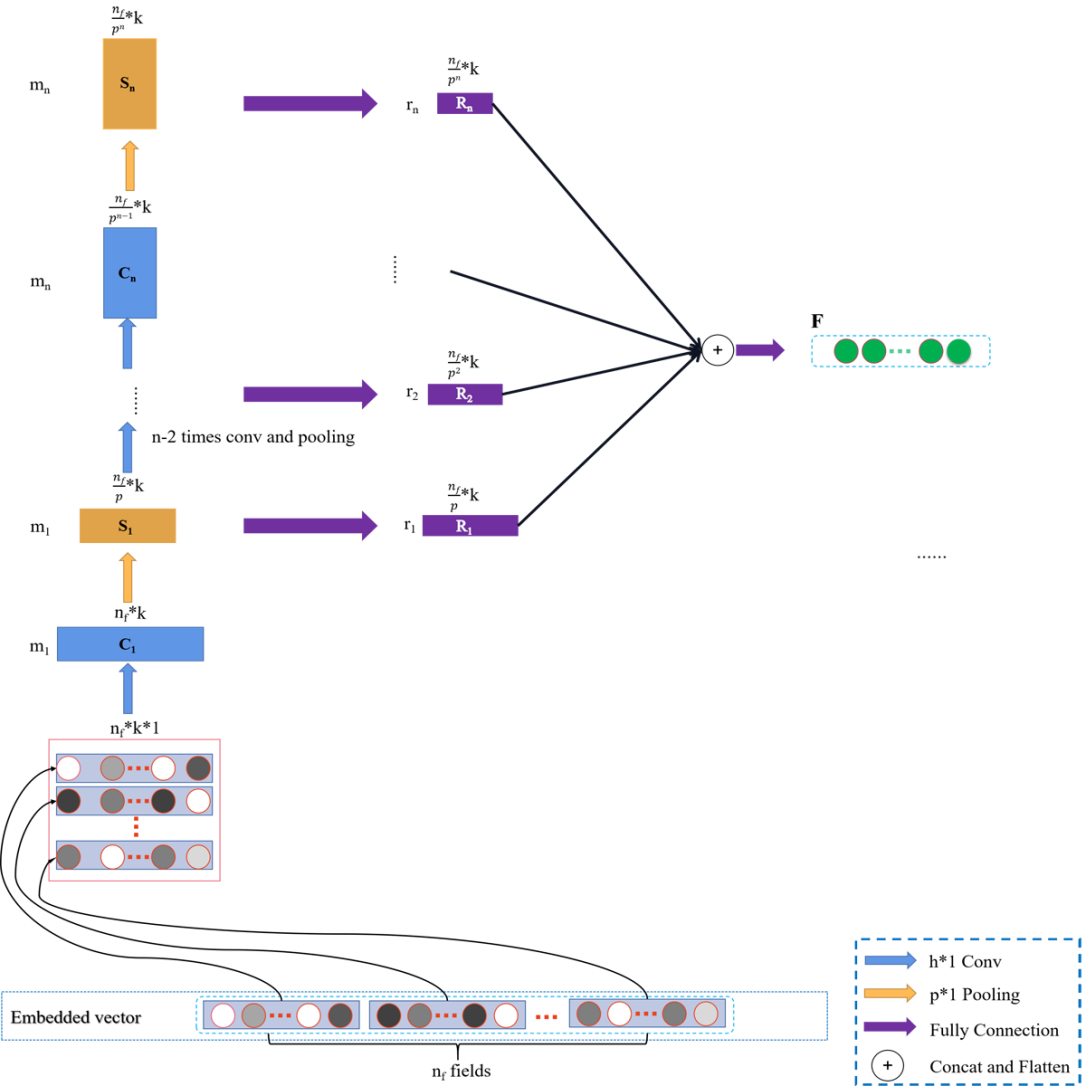
Improved feature generation by convolution neural network.

Due to the small number of parameters in the cross network, the ability of the model was limited. To capture high-order nonlinear crossover features, we introduced a deep network in parallel, as shown in Figure 6. It is difficult for the traditional neural network to learn local patterns; however, a convolution neural network can quickly obtain local patterns through convolution operation and combine it to generate a new model. Unlike text or image classification models, intelligent dialectical models have a local correlation in the input data. Therefore, after the convolution neural network output the cross features of the local patterns, it was input into an MLP model to obtain some global cross information. In the convolution layer, we entered the matrix E obtained by the embedded layer, which is an n_f_*k*1 matrix, where n_f_ is the number of field and k is the embedding size. Then, it was convoluted with a convolution kernel with the size of h*1*m_1_, and the corresponding convolution value was obtained by using tanh as the activation function. Because of the property of convolution, the convolution kernel using h*1 can obtain the cross features of adjacent h rows and output the feature graph of the specified number of channels. In terms of the first convolution, the number of channels in the convolution kernel was m_1_, the size of the output convolution matrix of the first convolution C_1_ was n_f_*k*m_1_. After obtaining the convolution matrix, the first pooled matrix S_1_ was obtained through the maximum pooling layer p*1. After it was pooled, the matrix size was as follows:



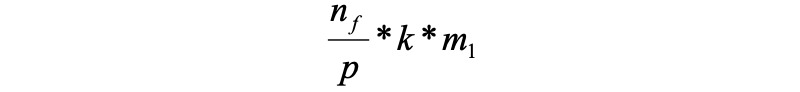



The pooled matrix S_1_ was passed down as the input of the next convolution layer and then recombined. Recombination was a fully connected operation shown as (3) and (4), using tanh as the activation function, B_i_ as the i_th_ recombination bias matrix, W_i_ as the i_th_ recombination weight matrix, and the first resulting high-order nonlinear feature size was as follows:



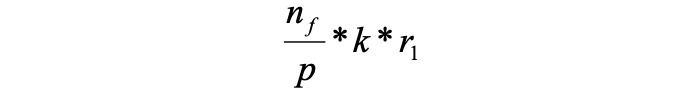



After this, repeat n-1 times convolution, pooling, and recombination, the entire convolution operation generated n reconstruction matrices, R= {R_1_, R_2_, …., R_n_}. To better integrate with the features of cross network output, R was converted into a new matrix according to the second dimension concat according to the traditional concept of convolution network, and then the reconstruction matrix flatten was passed into an MLP. Finally, a nonlinear high-dimensional feature vector F with local and global cross features was obtained.

R_i_ = tanh(S_i_W_i_+B_i_) (3)

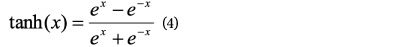



#### Classification Module

We combined the linear and nonlinear vectors constructed automatically by FGCNN and cross network to get the vector I_1_=(C,F), which is used as the input vector of the MLP. The input of the i_th_ hidden layer was O_i_, and its calculation is shown in (5), where W^i^ represents the weight matrix of the i_th_ hidden layer and B^i^ represents the bias matrix of the i_th_ hidden layer.

O_i_ =relu(I_i_W^i^+B^i^) (5)

Since there was a multiclassification problem, we made a final decision after the last hidden layer (n_h_):



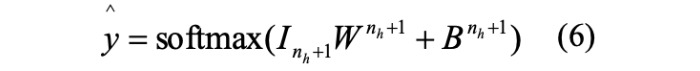



Finally, in the whole cross-FGCNN model, we chose cross-entropy as the loss function of the whole model.



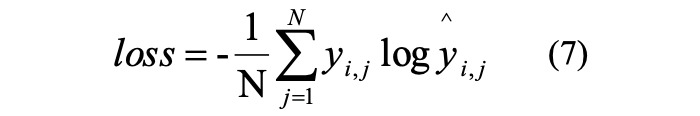



Where N was the total number of input data sets, Y was the real value, ŷ was the predicted value.

### Experiment

#### Experimental Operation

We used the cross-FGCNN and dysmenorrhea data mentioned earlier for the experiment. For training data, 75% of data were randomly selected and for test data, 25% of data were selected. A 60-dimensional label encoder vector was input, and 6-layer cross network was selected. In FGCNN, a convolution kernel with a depth of 14, 16, and 18 and a width of 4 was selected for a 3-layer convolution operation, and the depth of the MLP was 3 layers. A 3-layer neural network was selected in the classification module, and the number of neurons was 1024, 512, and 128. To maintain the robustness and optimization efficiency of the algorithm, we chose a dropout ratio of 0.2, set the learning rate to 0.001, and performed 1000 iterations.

#### Comparison With Other Models

A total of 6 traditional intelligent dialectical models were chosen: Bayesian classifier, multilabel K nearest neighbor (ML-KNN) classifier, 10-layer artificial neural network (ANN), decision tree, spectral clustering, and support vector machine. At the same time, to show the superiority of our algorithm for sparse symptom classification, 3 traditional CTR models DNN, FGCNN, and DCN were used in intelligent syndrome differentiation.

## Results

### The Experimental Results of Cross-FGCNN

Firstly, we calculated the model’s accuracy, which can directly express the reliability of the model.



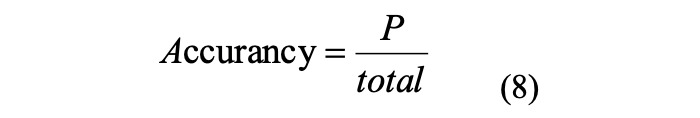



P represents the correct amount predicted by the model, and total represents the total number of input data from the model. The accuracy of cross-FGCNN was 96.21%. In Table 2, the amount of data between the classes of the entire data set was unbalanced, so we introduced F1 score and the confusion matrix [29].



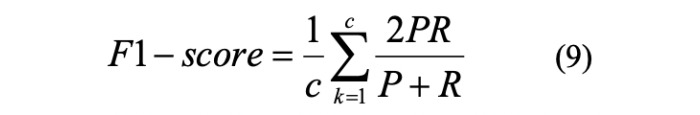



**Figure 7:**
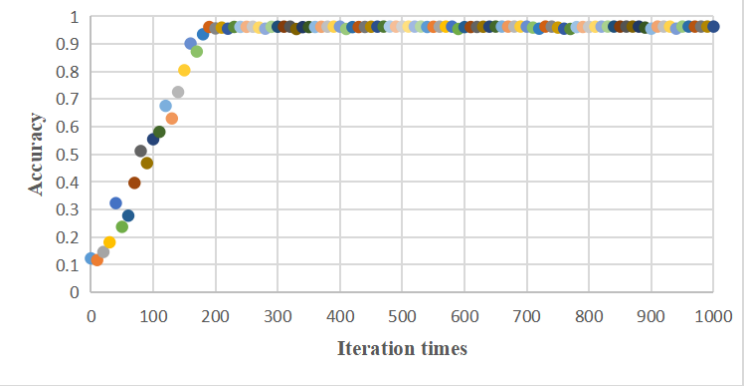
Cross-FGCNN accuracy-iteration times scatter diagram. FGCNN: feature generation by convolution neural network.

**Figure 8:**
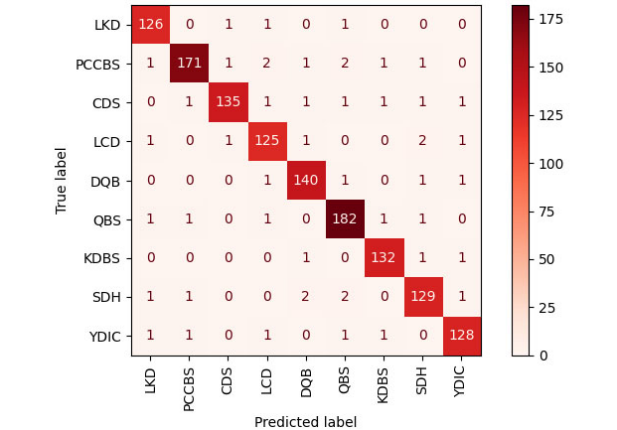
Cross-FGCNN confusion matrix. FGCNN: feature generation by convolution neural network; LKD: liver-kidney depletion; PCCBS: pattern of congealing cold with stasis; CDS: cold and dampness stagnation; LCD: liver constraint and dampness-heat; DQB: deficiency qi and blood; QBS: qi stagnation and blood stasis; KDBS: kidney deficiency and blood stasis; SDH: stagnation dampness-heat; YDIC: yang deficiency and internal cold.

P and R stand for precision and recall, respectively. The F1 score of cross-FGCNN was 0.9621, indicating that cross-FGCNN could be good at intelligent dialectics of TCM. Figure 7 shows the scatter diagram of the model accuracy changing with iteration times. After about 200 iterations, the accuracy of the model remained around 96%. Figure 8 shows cross-FGCNN’s classification confusion matrix, where the model divided all classes into the correct classes as much as possible. In short, cross-FGCNN showed great strength in intelligent dialectical tasks.

### Comparison Between Models

Table 3 shows the comparison between the cross-FGCNN model and other models with respect to accuracy and F1 score. Although 10-layer ANN [15] had a good classification effect, it still differed from the cross-FGCNN by 5% in accuracy. At the same time, the CTR model also displayed some potential in the intelligent dialectical task, where the accuracy of the FGCNN can even surpass the traditional intelligent, but there was still a gap compared with our model. To show how each model fits unbalanced classes, we introduced log-loss and receiver operating characteristic (ROC) curves.



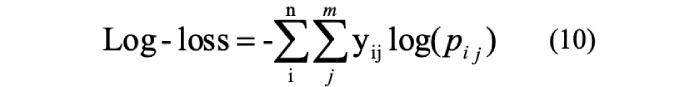



The equation represents the calculation of log-loss: n corresponds to the number of our samples or the number of inputted instances, i corresponds to a certain sample or instance, m represents the possible number of categories of our samples, j represents a certain category, and y_ij_ denotes that for a sample i, it belongs to the label of classification j. Therefore, the smaller the log-loss, the better the fitting effect of the reaction model, and cross-FGCNN still performed well in this respect. Compared with other models, cross-FGCNN manifested great potential for intelligent dialectics, which is a high-dimensional sparse vector multiclassification task.

**Figure 9:**
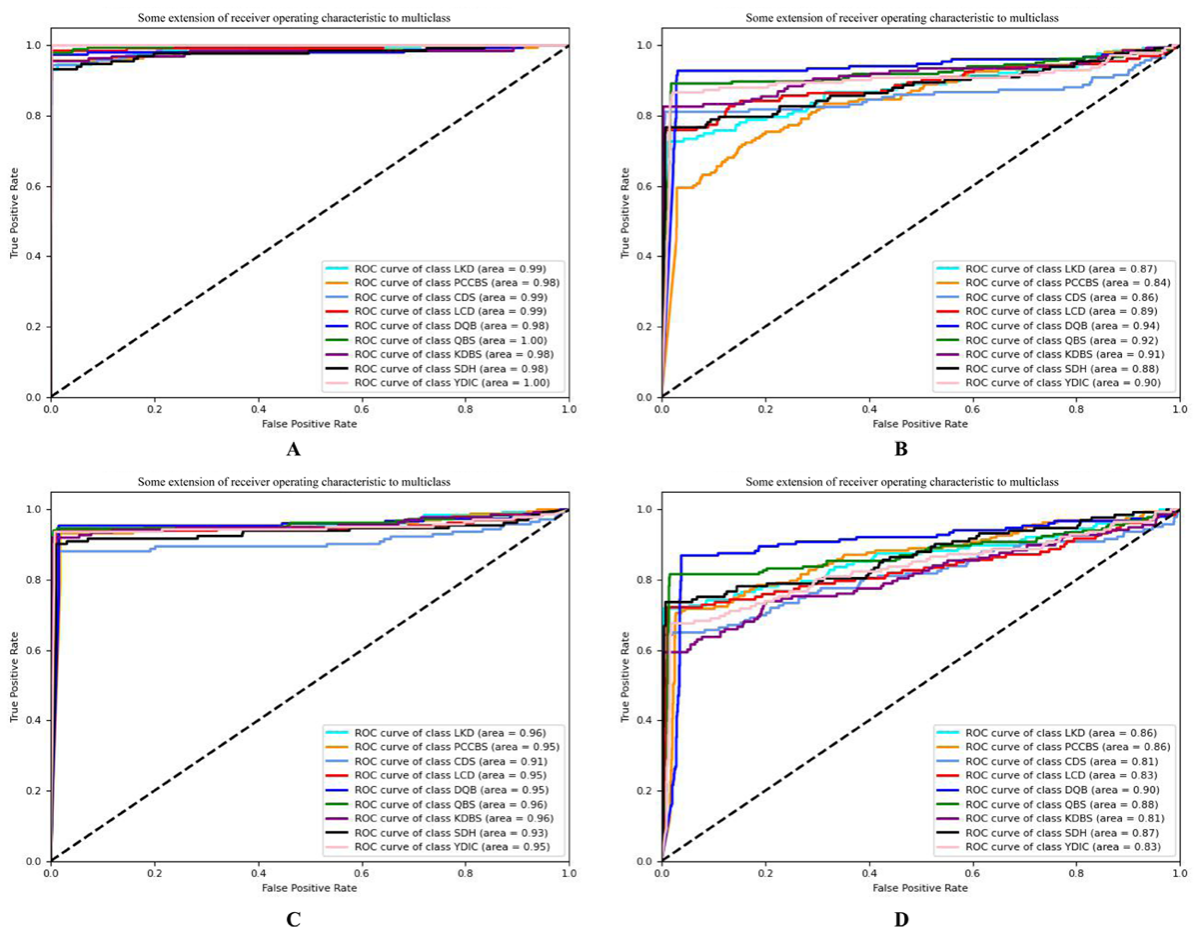
ROC Curves of CTR models. ROC: receiver operating characteristic; CTR: click-through-rate; FGCNN: feature generation by convolution neural network; deep neural network; LKD: liver-kidney depletion; PCCBS: pattern of congealing cold with stasis; CDS: cold and dampness stagnation; LCD: liver constraint and dampness-heat; DQB: deficiency qi and blood; QBS: qi stagnation and blood stasis; KDBS: kidney deficiency and blood stasis; SDH: stagnation dampness-heat; YDIC: yang deficiency and internal cold.

**Figure 10:**
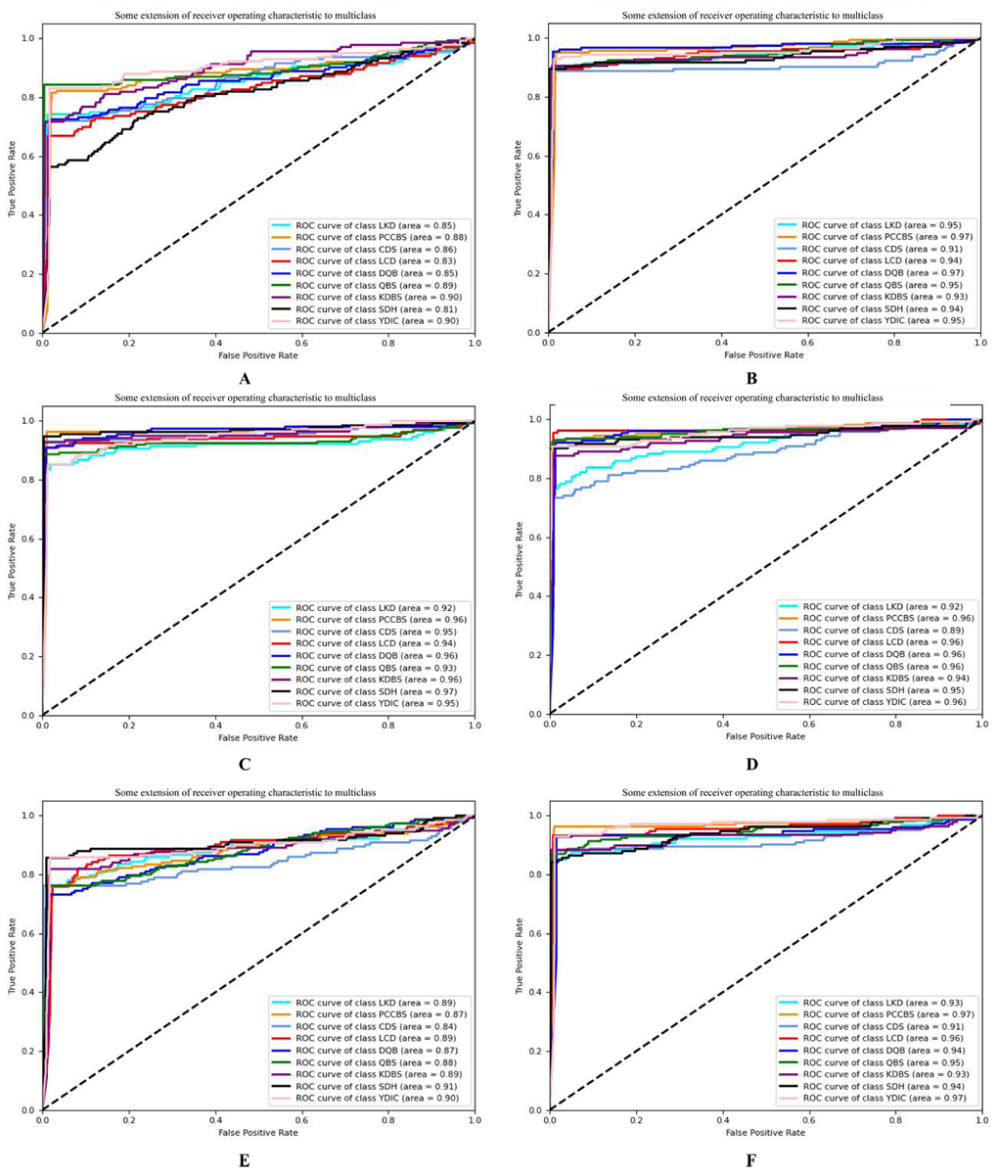
ROC curves of traditional intelligence dialectical models. ROC: receiver operating characteristic; ANN: artificial neural network; SVM: support vector machine; LKD: liver-kidney depletion; PCCBS: pattern of congealing cold with stasis; CDS: cold and dampness stagnation; LCD: liver constraint and dampness-heat; DQB: deficiency qi and blood; QBS: qi stagnation and blood stasis; KDBS: kidney deficiency and blood stasis; SDH: stagnation dampness-heat; YDIC: yang deficiency and internal cold; ML-KNN: multilabel K nearest neighbor.

The abscissa of the ROC curve was a false positive rate, and the ordinate was a true positive rate. The ROC curve remained constant as the distribution of positive and negative samples in the test set changed. For TCM syndrome differentiation, some syndrome types were rare. Therefore, it is necessary to evaluate the intelligent dialectical model by ROC. Intuitively, the closer the ROC curve was to the upper left corner, the better the model classification effect was. Figure 9 and Figure 10 show the ROC curve of a new model and the traditional CTR model and the traditional intelligent dialectical model, respectively. Figure 9A-Figure 9F respectively depicts the ROC curve of decision tree, 10-layer ANN, ML-KNN, hypergraph clustering, Bayesian, and SVM, and Figure 10A-Figure 10D respectively displays the ROC curve of cross-FGCNN, deep & cross network, FGCNN, and DNN. It is clear that cross-FGCNN outperformed the other models in the classification of different syndrome types. Secondly, the area under the ROC curve can also be used as one of the indicators of the model classification effect. By comparing the area under the macroaverage ROC curve, the new intelligent dialectical model still showed great strength. For the classification comparison of single syndrome type, it is obvious that cross-FGCNN outperformed the other models in syndrome differentiation.

**Table 3:** Result indicators of each model.

Model	Accuracy	F1 score	Log-loss
Cross-FGCNN^a^	0.9621	0.9621	0.8356
Decision tree	0.7448	0.7439	6.4533
10-layer ANN^b^	0.9121	0.9115	1.9071
ML-KNN^c^	0.9075	0.9076	2.7211
Hypergraph clustering	0.8816	0.8814	3.8436
Bayesian	0.7816	0.7815	4.5555
SVM^d^	0.8992	0.8989	3.2289
Deep & cross network	0.7992	0.7997	3.1602
FGCNN	0.9390	0.9390	1.2820
DNN^e^	0.7220	0.6804	3.9439

^a^FGCNN: feature generation by convolution neural network.

^b^ANN: artificial neural network.

^c^ML-KNN: multilabel K nearest neighbor.

^d^SVM: support vector machine.

^e^DNN: deep neural network.

## Discussion

TCM intelligent dialectic can be regarded as a classification model of a high-dimensional sparse vector. Based on this, we improved the new intelligent dialectical model with the CTR model. Different from other related studies, this study classified different symptoms into 60 fields under the guidance of TCM diagnostics. According to this method, the expected symptom information of the 4 diagnostic methods can correspond to different fields, achieve dimensionality reduction, and standardize symptom information. This method displayed strong portability because all fields were defined following the standard of TCM, and another data set could be made to construct a new system of syndrome differentiation and treatment. Furthermore, as proof, 5273 cases of dysmenorrhea were used to train cross-FGCNN in this study. In the model, cross network was used to construct new linear crossover vectors automatically, and FGCNN was used to construct new nonlinear crossover features. Two new features were combined for classification and compared with the other 9 types of models. Cross-FGCNN showed great potential in intelligent dialectics, a high-dimensional sparse vector multiclassification task. Nonetheless, advancements are still needed to achieve the overall optimization of the model and intelligent data acquisition.

1) The size of the data set has a great impact on the accuracy of the model, so data on dysmenorrhea and other diseases are still continuously collected to verify and improve the model.

2) The quality of data is also an essential factor affecting the model. Next, we not only continue to include new data but should also strictly check acquisition of new data and invite more professional practitioners of TCM to clean the data.

3) Intelligent medical treatment is the whole process of intelligence from data collection to patient prescription. Although we performed intelligent dialectics, it is still difficult to guarantee the reliability of data collection. There are still subjective judgments of TCM doctors in tongue diagnosis and other diagnoses, so we have begun to build an intelligent inspection model.

4) In the future, our team will construct an objective, TCM intelligent 4-diagnosis system, which integrates objective observation of TCM, intelligent listening of cough sound, remote intelligent consultation, intelligent acupoint detection of flexible portable equipment, and intelligent dialectics.

## Abbreviations

ANN: artificial neural network
CTR: click-through-rate
DCN: deep & cross network
DNN: deep neural network
FGCNN: feature generation by convolution neural network
FM: factorization machine
ML-KNN: multilabel K nearest neighbors
MLP: multilayer perceptron
SVM: support vector machine
TCM: traditional Chinese medicine
ROC: receiver operating characteristic
